# The Moderating Role of Coping Self-efficacy on the Relationship Between Stress and Psychosomatic Symptoms in Adolescents: A Moderation Analysis

**DOI:** 10.1192/j.eurpsy.2025.379

**Published:** 2025-08-26

**Authors:** M. Klein, D. Várnai, Á. Németh, G. Kökönyei

**Affiliations:** 1 Doctoral School of Psychology; 2 Institute of Psychology, ELTE Eötvös Loránd University; 3 Heim Pal National Institute of Pediatrics; 4 NAP3.0-SE Neuropsychopharmacology Research Group, National Brain Research Program; 5 Department of Pharmacodynamics, Faculty of Pharmacy, Semmelweis University, Budapest, Hungary

## Abstract

**Introduction:**

Adolescents face significant stress, which can undermine their subjective health, often manifesting as psychosomatic symptoms. Perceived self-efficacy is a critical resource for resilience, potentially buffering the impact of stress on subjective health. While action self-efficacy indicates one’s perceived ability to set goals and take initiative, coping self-efficacy refers to one’s ability to achieve the goals. By investigating both components, researchers and mental-health professionals can better understand how different aspects of self-efficacy influence the stress-symptom relationship, providing a more nuanced view of coping mechanisms.

**Objectives:**

The aim of this study is to determine how these key components of self-efficacy - action self-efficacy and coping self-efficacy - moderate the relationship between stress and psychosomatic symptoms.

**Methods:**

The analyses were based on the Hungarian contribution to the representative international Health Behaviour in School-aged Children (HBSC) survey, collected in 2021/2022. The study population comprised self-report data from high-school students (N = 3,410; mean age = 16.77 years). We examined the main effects of self-efficacy dimensions and perceived stress, and their interactions in explaining psychosomatic complaints, using linear regression analysis with Hayes’ PROCESS macro (Model 1) in SPSS. Age and gender were controlled for as covariates.

**Results:**

*Coping self-efficacy*: The overall model explained 33% of the variance in psychosomatic symptoms (R² = 0.33, F(5, 3404) = 330.96, p < .001). The results showed that stress (b = 1.93, p < .001), coping self-efficacy (b = 2.18, p = .02), and their interaction (b = -0.33, p = .002) were significant explanatory variables. This suggests that the relationship between stress and psychosomatic symptoms is weaker among adolescents who reported stronger coping self-efficacy. *Action self-efficacy*: In contrast, the interaction between stress and action self-efficacy was not significant (p = .16), implicating that the ability to set goals did not mitigate the effect of stress on symptoms.

**Conclusions:**

The findings imply that coping self-efficacy significantly reduces the relationship between stress and psychosomatic symptoms. Adolescents with stronger coping skills are better equipped to mitigate the adverse effects of stress. However, action self-efficacy did not show a significant moderating effect, highlighting the distinct roles different self-efficacy components play in stress management. These results emphasize the importance of enhancing coping skills, such as cognitive reappraisal, in primary prevention interventions to reduce stress-induced psychosomatic symptoms.

**Keywords:**

adolescents, HBSC, psychosomatic symptoms, self-efficacy, stress

This study was supported by the Hungarian National Research, Development, and Innovation Office (K139265 and K143764).

**Disclosure of Interest:**

None Declared

